# Recurrent heart failure with preserved ejection fraction associated with carfilzomib administration for multiple myeloma

**DOI:** 10.1186/s40959-018-0028-z

**Published:** 2018-03-01

**Authors:** Eric H. Yang, Cynthia Courtney, Vinisha Garg, Michael G. Fradley, Gary J. Schiller

**Affiliations:** 1 UCLA Cardio-Oncology Program, Division of Cardiology, Department of Medicine UCLA Cardiovascular Center, 100 Medical Plaza, Suite 630, Los Angeles, CA 90095 USA; 2 Division of Hematology and Oncology, David Geffen School of Medicine at UCLA, Los Angeles, CA USA; 3 Cardio-oncology Program, Department of Cardiovascular Sciences, University of South Florida Morsani College of Medicine and H. Lee Moffitt Cancer Center and Research Institute, Tampa, FL USA

**Keywords:** Carfilzomib, Proteasome inhibitors, Multiple myeloma, Heart failure, Cardiac biomarkers

## Abstract

Carfilzomib, an epoxyketone proteasome inhibitor, has demonstrated improved progression-free survival in patients when used with standard treatment (lenalidomide and dexamethasone) in patients with relapsed multiple myeloma (MM). However, there are reports of adverse cardiac events with carfilzomib manifested by dyspnea and heart failure. A patient is presented who had recurrent, clinically mild cardiotoxicity, as manifested by recurrent heart failure with preserved ejection fraction, with ongoing maintenance carfilzomib in a patient with resistant MM is presented.

## Background

Multiple myeloma (MM) comprises about 1% of neoplastic diseases and 13% of hematologic disorders. With advances made in autologous stem cell transplantation and medical treatment, clinical response and survival rates have improved. [1, 2]. Carfilzomib, an epoxyketone proteasome inhibitor, has demonstrated improved progression-free survival in patients when used with standard treatment in patients with relapsed MM [3]. In July 2015, the United States Food and Drug Administration approved carfilzomib, 27 mg/m^2^ days 1, 2, 8, 9, 15, and 16 (20 mg/m^2^ on days 1, 2 of cycle 1) on a 28-day cycle for usage in combination with standard-dose lenalidomide and dexamethasone for the treatment of patients with relapsed MM who had received 1–3 prior lines of therapy [4]. Reports of adverse cardiac events with carfilzomib included, documented symptoms and manifestations of cardiotoxicity including dyspnea and heart failure. We present a patient with relapsed MM treated on a Phase Ib clinical trial of carfilzomib who had recurrent, clinically mild heart failure with preserved ejection fraction (HFpEF)—as demonstrated by biomarkers and invasive hemodynamics.

A 58 year old female with monoclonal IgG lambda MM presented with onset of dyspnea following treatments with carfilzomib. Her hematologic history was significant for MM diagnosed 7 years prior through routine blood work. Despite five years of treatment, she developed progressive myeloma and ultimately underwent autologous stem cell transplant. She again developed progression of her myeloma requiring advanced clinical therapy. Her other past medical history was remarkable only for dyslipidemia. Her medications consisted of aspirin 81 mg daily, atorvastatin 20 mg daily, acyclovir 400 mg twice daily, pantoprazole 40 mg daily, venlafaxine 75 mg daily, ascorbic acid 1 g daily, calcium carbonate 600 mg daily, Vitamin D and tumeric supplements. Her family history was negative for premature coronary artery disease or sudden death. She quit tobacco use over 25 years prior and denied any significant alcohol or illicit drug use. With regards to her chemotherapy treatments, she initially underwent treatment with cyclophosphamide, lenalidomide, and dexamethasone, and subsequently continued on lenalidomide and dexamethasone for maintenance. After two years of treatment, she developed progressive myeloma and was initiated on lenalidomide, bortezomib and dexamethasone, resulting in morphologic and cytogenetic remission. She then underwent autologous stem cell transplant and received 2 years of lenalidomide maintenance therapy.

One year prior to presentation, her M protein levels began to rise along with evidence of a new osteolytic lesion with a rib fracture on bone survey imaging. She subsequently enrolled in a phase Ib clinical trial of intravenous carfilzomib 70 mg/m2 (approximately 112 mg) administered once weekly for 3 weeks out of a 4-week cycle, in combination with oral lenalidomide 25 mg daily for 21 days per cycle, and oral dexamethasone 40 mg weekly. She had no cardiac complications or symptoms during previous treatment.

Approximately 1 month into treatment, she began noticing reproducible complaints of chest tightness and pressure with occasional associated palpitations, which she experienced within a couple hours after infusion of carfilzomib. These symptoms would occur with mild physical exertion, although this was not outright limiting her functionality. She denied any orthopnea, lower extremity edema, presyncope or syncope during these episodes. After 2–3 days, these episodes would resolve without intervention. During the 4th week of the cycle when no infusion was given, she was asymptomatic. She was referred to cardiology for evaluation of these recurrent episodes.

At time of initial cardiology evaluation, she was asymptomatic and her exam showed an afebrile patient with a temperature of 37.2 degrees Celsius, heart rate of 97 beats per minute, blood pressure of 109/63, respiratory rate of 18, oxygen saturation of 97% at room air. The patient was alert and oriented and in no acute distress. Cardiac examination was negative for any murmurs, rubs, or S3/S4 gallops. Her jugular venous pressure was approximately 6 cm H20. Lungs were clear to auscultation. No peripheral edema was observed with normal distal pulses. Her physical examinations did not significantly vary throughout her treatment course, both before and after her infusions.

**Fig. 1:**
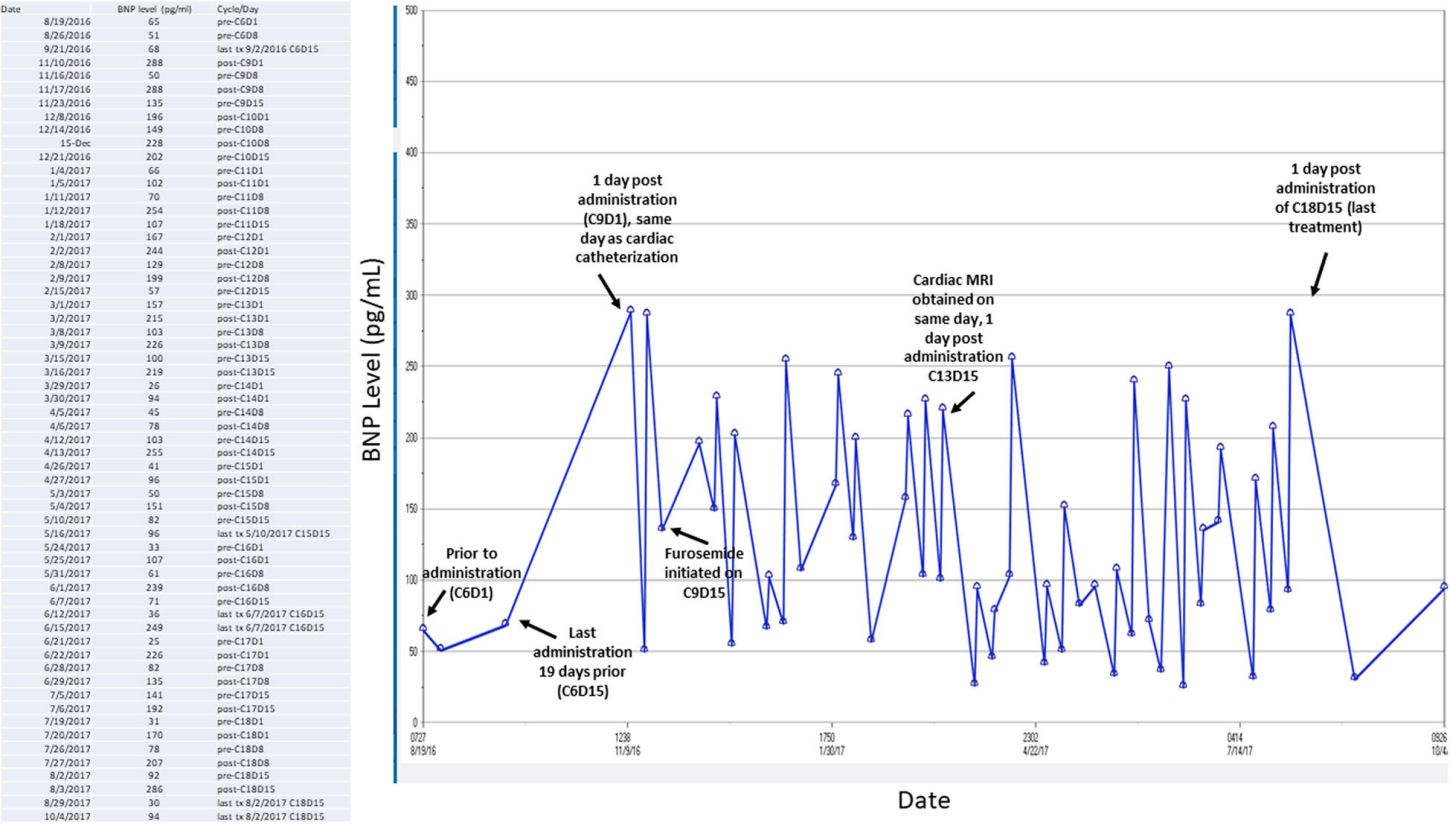
B-natriuretic peptide (BNP) biomarker levels obtained during course of carfilzomib treatment. Prior to carfilzomib infusion on cycle 9, day 8, BNP levels normalize with no intervention despite having elevated levels the week prior with a sharp increase with identical levels the day after. On the 15th day of treatment, prior to administration, BNP levels are noted to be elevated. On C9D15, oral furosemide 20 mg/day was initiated to be taken only on the day of the patient’s carfilzomib infusions, which resolved the patient’s symptoms. During cycle 10, it is noted that even before treatment, BNP levels are noted to be elevated, although the patient was asymptomatic. BNP levels obtained on “pre-” days are on same day of treatment, right before infusion. BNP levels obtained “post-” are drawn 1 day after infusion. C: Cycle, D: day. MRI: magnetic resonance imaging. Normal value is < 100 pg/ml

Laboratory results on initial evaluation demonstrated a white blood cell count of 3.19X103/μL, hemoglobin of 9.6 g/dL, platelet count of 99X103/μL. A sodium level was 144 mmol/L, potassium 4.6 mmol/L, chloride 106 mmol/L, bicarbonate level 25 mmol/L, blood urea nitrogen 13 mg/dL, creatinine 0.90 mg/dL, uric acid 4.2 mg/dL, and a lactate dehydrogenase of 203 U/L. Liver function testing was unremarkable. B-natriuretic peptide (BNP) serum biomarker was normal. BNP levels were serially assessed throughout her treatment course, and were obtained predominantly 1 day before and after infusion. They demonstrated predominantly normal levels just prior to carfilzomib infusion; however, 1 day post infusion, recurrent biomarker elevations were noted as high as 288 pg/mL (normal < 100 pg/mL) (Fig. 1). In most cases, levels would normalize without any intervention performed the following week. Typically, 250 mL of intravenous normal saline would be administered with her carfilzomib.

**Fig. 2:**
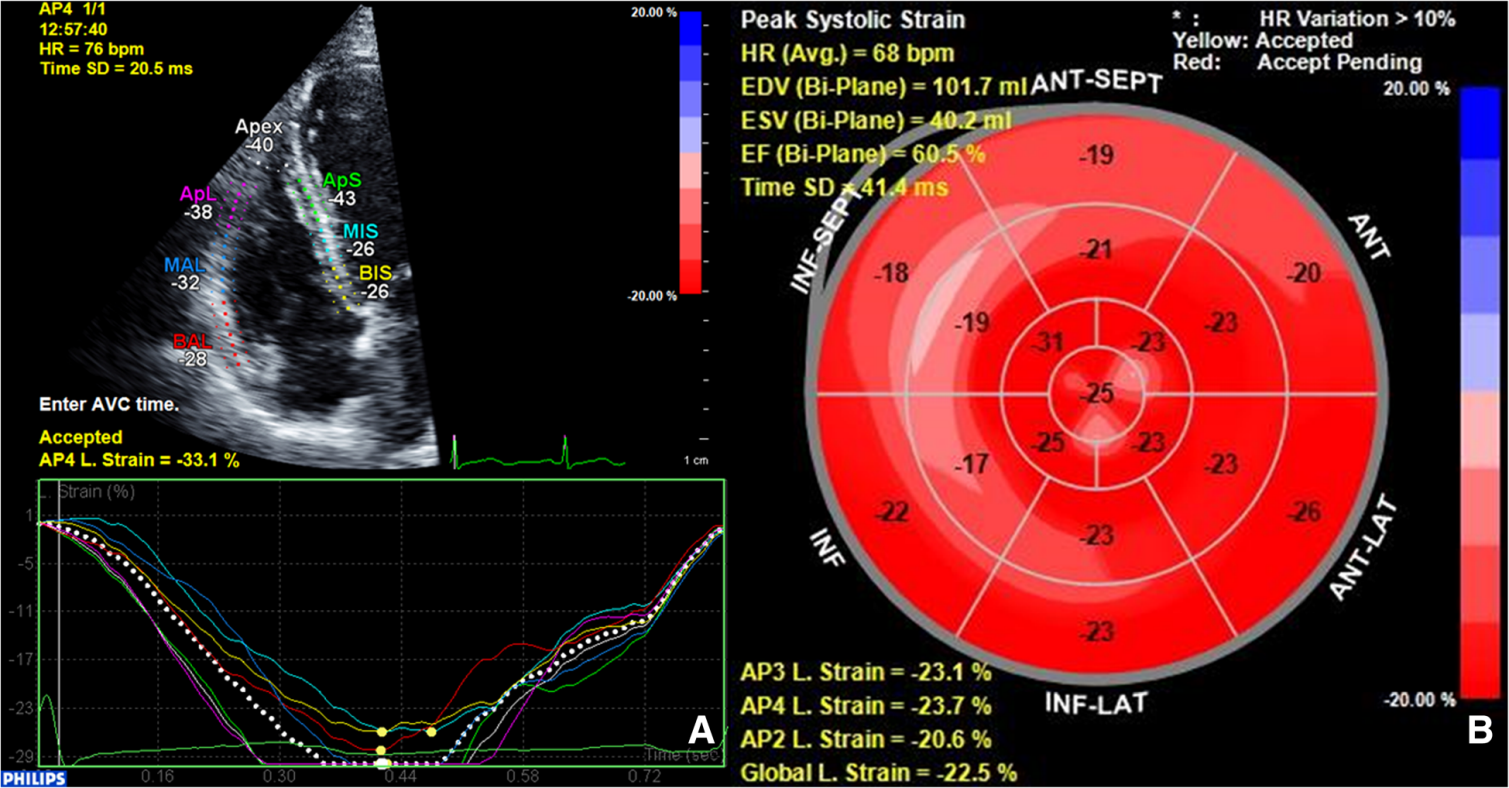
Speckle tracking and strain measurements performed on transthoracic echocardiogram performed approximately 1 h after carfilzomib administration on the 1st day of 6th cycle of treatment. Panel (**a**): Speckle tracking measurements performed of the right ventricle with a longitudinal strain rate of − 33.1% (normal for age, gender and software). Panel (**b**): Polar map of left ventricular strain measurements of transthoracic echocardiography Speckle tracking measurements estimated a biplanar LV ejection fraction of 61% with normal strain values with a global longitudinal strain of − 22.5% (normal for age, gender and software)

**Fig. 3:**
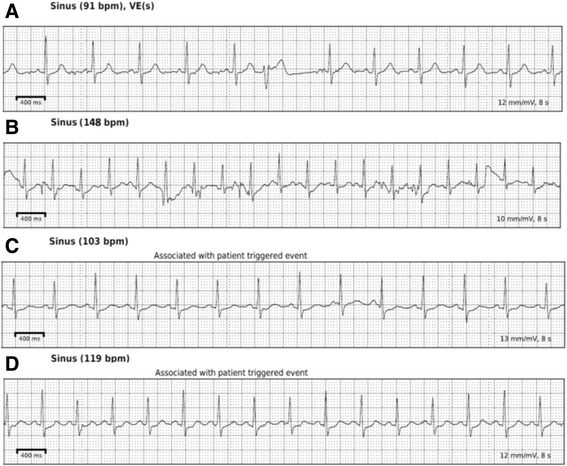
Rhythm tracings from 2 week continuous rhythm monitoring with a Zio patch (iRhythm, San Francisco, CA). Panel (**a**): Baseline asymptomatic recording demonstrating sinus rhythm with 1 ventricular ectopic beat. Normal ST segments are seen. Panel (**b**): Asymptomatic recording during sinus tachycardia at the highest heart rate documented during entire recording. Motion artifact is noted but overall no abnormal ST-T segments noted. Panel (**c**): Patient triggered event due to symptoms, demonstrating sinus tachycardia with approximately 0.5 mm ST-T segment depressions. The patient received carfilzomib earlier that day (during 6th cycle of treatment). Panel (**d**): Patient triggered event with symptoms 1 day after 6th cycle of treatment (day of Panel C) demonstrating sinus tachycardia with 0.5–1 mm ST-T segment depressions noted with symptoms

**Fig. 4:**
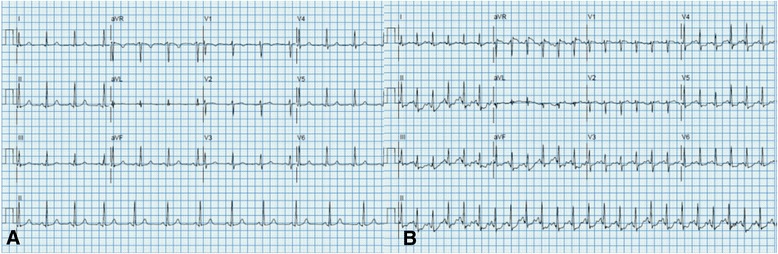
Electrocardiographic (ECG) tracings of exercise echocardiogram performed 8 days after the 3rd dose of carfilzomib administration of the 5th cycle of treatment. Panel (**a**): ECG tracing at rest showing normal sinus rhythm with no significant ST-T segment abnormalities or axis deviation. Panel (**b**): ECG tracings at peak exercise (90% maximum predicted heart rate, 7.0 METs) demonstrating approximately 1 mm ST-T segment depressions in inferolateral leads which resolve in recovery. Patient also experienced symptoms similar to her prior dyspnea at peak exercise. Echocardiographic portion of exam showed no exercise induced wall motion abnormalities at peak exercise

A 12-lead electrocardiogram (ECG) demonstrated normal sinus rhythm with no abnormal ST-T segments. A corrected QT interval was 446 msec. A chest radiograph was unremarkable with no evidence of pulmonary edema or cardiac silhouette enlargement. Serial transthoracic echocardiography (TTE) obtained approximately 1 h post carfilzomib infusion demonstrated normal LV and RV function with normal strain values (Fig. 2). No significant valvular regurgitation or pulmonary hypertension was noted. Two-week continuous rhythm monitoring did not demonstrate any tachyarrhythmias, but ST-T segment depressions were associated with symptoms of dyspnea and palpitations (Fig. 3). Exercise echocardiography was performed 8 days after her last infusion during her 5th cycle of treatment, where the patient reached 7.0 METs and 90% maximum predicted heart rate. At peak exercise, 1 mm ST-T segment depressions were noted with symptoms similar to her dyspnea (Fig. 4) with a normal echocardiographic portion. A coronary computed tomography angiogram was performed showing mild calcified plaque in the proximal left anterior descending artery (LAD) with subsequent superficial myocardial bridging in the mid-LAD segment. At that point, low-dose metoprolol tartrate was prescribed with no improvement in her symptoms.

**Fig. 5:**
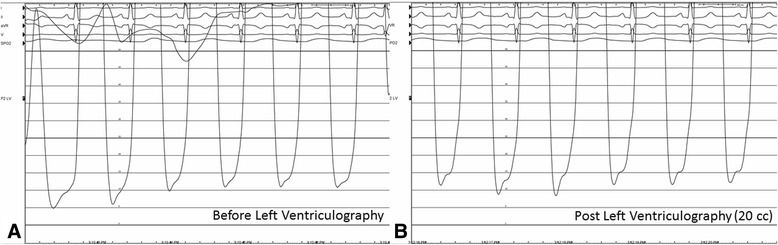
Pressure waveforms measured in the left ventricle during left cardiac catheterization with a 5 French pigtail catheter. Procedure was performed 1 day after the first treatment on the 9th cycle of carfilzomib administration. Panel (**a**): Measurements obtained prior to left ventriculography with an end expiratory left ventricular end diastolic pressure (LVEDP) of approximately 18 mmHg, and a left ventricular systolic pressure was approximately 134 mmHg. Panel (**b**): Measurements obtained immediately after 20 cm^3^ of iodinated contrast of left ventriculography with an increase of the LVEDP to 22 mmHg, with a left ventricular systolic pressure of 142 mmHg. LV ejection fraction was approximately visualized at 50–55% but hampered by significant ventricular ectopy during ventriculography. BNP level measured after procedure was elevated at 288 pg/ml

To exclude macrovascular disease and to evaluate cardiac hemodynamics, a left heart catheterization was performed 1 day after her 9th cycle of carfilzomib treatment revealing no significant epicardial coronary artery disease. Her left ventricular end diastolic pressures were noted to be elevated at 18 mmHg which increased post ventriculography to 22 mmHg. (Fig. 5); her BNP level on the same day as 288 ng/mL. Cardiac magnetic resonance imaging performed 1 day after day 15 of her 13th treatment cycle demonstrated an LV ejection fraction of 58% with normal LV and RV systolic function. No abnormal delayed enhancement in the myocardium was seen. Her BNP level on the same day was elevated at 219 ng/mL.

Following her catheterization findings, she was instructed to take furosemide 20 mg on the day of her infusion, which resulted in near resolution of her symptoms. Upon initial evaluation, she was administered 25 mg metoprolol tartrate twice daily, which did not improve her symptoms. It was stopped 3 months later, after it was found that furosemide improved her symptoms more effectively. Discussions in weighing risks and benefits of continuing treatment were held and given she was demonstrating clinical response to her MM, and she had a relatively stable cyclical trend of her biomarkers and symptoms, it was decided that she continue treatments with close biomarker, imaging, and clinical surveillance. She continues on carfilzomib treatments to this day.

## Discussion

Carfilzomib in phase II single-agent studies yielded adequate safety outcomes [5] and also demonstrated progression-free survival in combination with dexamethasone and lenalidomide compared to dexamethasone and lenalidomide alone in the ASPIRE trial [3]. However, cardiac adverse events were reported, with most manifestations presenting as cardiac failure (6.4% of patients on carfilzomib) [3]. Symptoms were generally more common within the first 18 cycles of treatment than in later cycles [3]. In an overall analysis of adverse event of 4 phase II studies (*n* = 526) in patients with advanced MM, 42.2% reported dyspnea with treatment and 7.2% developed heart failure [5]. Patients in these trials had carfilzomib intravenously administered on days 1,2,8,9, 15, and 16 in 28 day cycles with a typical dose of 20 mg/m2 on cycle 1 with escalation to 27 mg/m2 in cycle 2 in most studies. In addition, the ASPIRE trial also showed a higher incidence of heart failure, ischemic heart disease, hypertension and venothromboembolic disease in the carfilzomib group compared to the control group [5].

The degree of heart failure has been described in various degrees of severity, and has been characterized by LV systolic dysfunction with echocardiographic indices of diastolic dysfunction, although heart failure with preserved ejection fraction is uncommon. [6, 7]. Mechanisms of how cardiotoxicity is caused is unclear, but theoretical mechanisms include pro-apoptotic actions via induction of capase-3/7, as well as inhibition of the chymotrypsin-like activity of myocyte lysate, which contributes to protein turnover in the heart [8] and possible endothelial dysfunction [9]. Biomarkers such as NT-proBNP have been shown to significantly increase after carfilzomib administration; however, imaging assessments have reported varying results including normal systolic and diastolic function but abnormal LV longitudinal strain. [6, 9–11].

The course of this patient’s treatment with recurrent dyspnea following carfilzomib treatments was unique given the consistent reproducibility of her symptoms immediately following her infusions, which would wax and wane with her biomarker trends. Most cases of suspected carfilzomib induced heart failure in the literature were not rechallenged, with a minority of cases undergoing a lower dose of carfilzomib infusions afterwards [6, 7]. The patient also did not have any significant risk factors for cardiovascular disease—which was also confirmed on invasive coronary angiography--with only dyslipidemia and a remote tobacco history. In this patient, it was thought that carfilzomib had induced transient, recurrent HFpEF physiology as manifested by ischemic ECG changes without macrovascular disease, and elevated BNP levels. This was confirmed invasively with a left heart catheterization 1 day post infusion with elevated left ventricular filling pressures; her symptoms would mostly or completely normalize along with her symptoms within several days. Adding furosemide to the patient’s regimen improved her symptoms likely by reducing her ventricular preload, but was not needed on a maintenance dose due to the transient nature of these symptoms. It is also notable that the patient received weekly higher doses of carfilzomib compared to prior MM clinical trials.

In summary, we present a case of carfilzomib-induced cardiotoxicity that manifested as recurrent symptoms of dyspnea with preserved cardiac function, but with abnormal biomarker elevations following each administration of carfilzomib—which correlated to high left ventricular filling pressures—that would return to normal or near normal levels prior to the next administration. Administration of furosemide allowed for alleviation of symptoms, with eventual normalization of BNP levels. This case is particularly unique in that it highlights the possibility of cardiotoxicity from carfilzomib within a few weeks of treatment in relatively young patients with low cardiovascular risk. Further studies and research are needed to investigate diagnostic modalities in identifying cardiotoxicity with carfilzomib, as well as investigating cardioprotective strategies to attenuate risk in developing cardiac adverse outcomes.

## Abbreviations

BNP: B-natriuretic peptide
ECG: Electrocardiogram
HFpEF: Heart Failure with Preserved Ejection Fraction
LAD: Left anterior descending artery
LV: Left ventricular
MM: Multiple myeloma
RV: Right ventricular
TTE: Transthoracic Echocardiogram

## References

[1] Palumbo A, Anderson K (2011). Multiple Myeloma. N Engl J Med.

[2] Moreau P, Richardson PG, Cavo M (2012). Proteasome inhibitors in multiple myeloma: 10 years later. Blood.

[3] Stewart AK, Rajkumar SV, Dimopoulos MA (2015). Carfilzomib, Lenalidomide, and dexamethasone for relapsed multiple myeloma. New Engl J Med.

[4] FDA approval for Carfilzomib/Kyprolis. https://www.accessdata.fda.gov/drugsatfda_docs/appletter/2016/202714Orig1s010ltr.pdf. Last accessed 16 Feb 2018.

[5] Siegel D, Martin T, Nooka A (2013). Integrated safety profile of single-agent carfilzomib: experience from 526 patients enrolled in 4 phase II clinical studies. Hematologica.

[6] Grandin EW, Ky B, Cornell F, Carver J, Lenihan DJ (2015). Patterns of cardiac toxicity associated with irreversible proteasome inhibition in the treatment of multiple myeloma. J Card Failure.

[7] Li W, Cornell F, Lenihan D (2016). Cardiovascular complications of novel multiple myeloma treatments. Circulation.

[8] Hasinoff BB, Patel D, Wu X. Molecular mechanisms of the cardiotoxicity of the proteasomal-Targted drugs Bortezomib and carfilzomib. Cardiovasc Toxicol. 2016; 10.1007/s12012-016-9378-7. 10.1007/s12012-016-9378-7 27388042

[9] Rosenthal A, Luthi J, Behlolavek M (2016). Carfilzomib and the cardiorenal system in myeloma: an endothelial effect?. Blood Cancer J.

[10] Atrash S, Tullos A, Panozzo S (2015). Cardaic complications in relapsed and refractory multiple myeloma patients treated with carfilzomib. Blood Cancer J.

[11] Chari A, Hajje D (2014). Case series discussion of cardiac and vascular events following carfilzomib treatment: possible mechanism, screening and monitoring. BMC Cancer.

